# An Embodied Tutoring System for Literal vs. Metaphorical Concepts

**DOI:** 10.3389/fpsyg.2018.02254

**Published:** 2018-11-28

**Authors:** Marietta Sionti, Thomas Schack, Yiannis Aloimonos

**Affiliations:** ^1^Neurocognition and Action Research Group-Biomechanics, Cluster of Excellence-Cognitive Interaction Technology, Bielefeld University, Bielefeld, Germany; ^2^Faculty of Psychology and Sports Science, Bielefeld University, Bielefeld, Germany; ^3^Computer Vision Laboratory, Department of Computer Science, University of Maryland, College Park, MD, United States

**Keywords:** motion capture data, educational (serious) game, literal meaning, metaphors, language teaching, spatiotemporal memory

## Abstract

In this paper we combine motion captured data with linguistic notions (preliminary study) in a game-like tutoring system (study 1), in order to help elementary school students to better differentiate literal from metaphorical uses of motion verbs, based on embodied information. In addition to the thematic goal, we intend to improve young students’ attention and spatiotemporal memory, by presenting sensorimotor data experimentally collected from thirty two participants in our motion capturing labs. Furthermore, we examine the accomplishment of tutor’s goals and compare them to curriculum’s approach (study 2). Sixty nine elementary school students were randomly divided in two experimental groups (game-like and traditional) and one control group, which did not undergo an intervention. All groups were tested in pre and post-tests. Even though the diagnostic pretests present a uniform picture, two way analysis of variance suggests that the experimental groups showed progress in post-tests and, more specifically, game-like group showed less wrong answers in the linguistics task and higher learning achievements compared to the other two groups. Furthermore, in the game-like condition the participants needed gradually shorter period of time to identify the avatar’s actions. This finding was considered as a first indication of attentional and spatiotemporal memory’s improvement, while the tutor’s assistance features cultivated students’ metacognitive perception.

## Introduction

Several disciplines dictate the necessity of investigating the embodied accounts of cognition (Stojanov, 1999; Barsalou, 2008; Fischer and Zwaan, 2008; Kövecses, 2015; Jamrozik et al., 2016) whether sensorimotor features shape the mechanism of semantic structure or not. Piaget (1952) psychology claims that the first data perceived and processed in infancy are of sensorimotor nature. For this reason Piaget named the first developmental period as the sensorimotor stage of cognitive development. On the other hand, Mandler (2004) considers that children apart from sensorimotor experiences, they develop rich concepts of universal nature. Mandler (1999, 2010, 2012) and Mandler and Cánovas (2014) suggests the existence of *spatial primitives* as conceptual building blocks, which build spatial stories, the *image schemas*. These two notions –primitives and schemas- integrate and create concepts that include every conceptual element. Concerning *images schemata* Johnson (1987) and Lakoff and Johnson (2003) further analyze them as more objective representations of the surrounding natural world, while their verbal description is a different symbolic system and it lacks full metacognitive description of the *image schemas*’ nature. Their suggestions –and their consequent *neural theory of metaphor* (Narayanan, 1997; Bergen, 2011) embrace the neurological findings of Rizzolatti et al. (1996), who allege that mirror neurons incorporate action in a sensorimotor form/gestalt. Due to these data, human brain forms concepts and expresses them through language. Abstract ideas –such as metaphors- are understood through concrete concepts and their associated sensorimotor simulations, e.g., words referring to literal actions strongly engage the somatotopic organization of motor and premotor cortex (Hauk et al., 2004; Gallese and Lakoff, 2005; Kemmerer et al., 2008; Boulenger et al., 2009; Lacey et al., 2012). Even researchers that suggest a weeker embodied account of cognition (Boroditsky, 2000; Boroditsky and Ramscar, 2002; Chatterjee, 2010; Cardillo et al., 2012) accept the importance of sensorimotor information in comprehending metaphors.

Based on these assumption, we presume that, even though children already possess action image schemas that correspond to motion verbs, e.g., *turn around*, they are not fully able to recognize the prototypical action depicted by verbs. Moreover, they have not fully associated the action-verb pair with literal concept and through literacy to discern metaphorical meaning. Children still use intuitive means to distinguish literacy from metaphor, which are still unstable and cause mistakes. Instead, if they concretely see the action and immediately associate it with literal vs. metaphorical use, they realize it as fundamental metacognitive technique that can be used afterward without the stimulus but by mental recall.

In the remaining of the paper, we present why we selected 3D data for our tutoring system and how we collected these Motion Capture data (preliminary study). Then we explain the fundamentals of Intelligent Tutoring Systems (ITS) and serious gaming principles with pedagogical objectives, based on which we propose our tutor’s architecture (study 1). Finally, we evaluate its learning effectiveness in elementary school students (study 2).

### Sensory Data Analysis

According to the communication model (Akmajian et al., 2001), the perception of a person’s movements and intentions -by another person that acts as receiver- is inextricably linked to the sense of vision. Darwin (Keltner and Gross, 1999: p. 4) acknowledged that gestures speak louder than pictures when it comes someone to understand what others do or feel. The unbroken unity of sensory (visual) and kinetic process is dictated by human movement’s perception itself, which depends on information collected from multiple sources. Besides, before the development of computer vision’s methods, researchers isolated human kinematics by using point-light display. Point-light exhibits biological activity represented by small lights arranged in large parts of the actor’s body. Optical analysis and determination of the human form through representations of biological movement as point-light have deep historical roots. The French physician Marey (1884) developed the chronophotography, which shows many shots in one photograph, while the actor was wearing black clothes with a small bright marker on each joint. Thus, utilizing the after-image of the human retina, chronophotography eliminated unnecessary elements and focused on joint ankles. This method was modified and developed in terms of individual details and techniques, but the core methodology remained stable until motion capture technology and computer animation came into rise. Successive images with point lights help observers understand the performed action even between subtle cases (Pinto and Shiffrar, 1999; Vanrie and Verfaillie, 2004). Troje et al. (2005) found that observers distinguish the actor’s gender based only on the observation of their movement.

Researchers began to complicate the process of masking point lights or the space-time distortion, in order to reveal subtle variations that constitute the difference of movements and to define the minimum properties of motion, through minimum stimulus. In the first joint masking technique, Pinto and Shiffrar (1999) found that the middle joints, such as elbows, knees, shoulders and hips may lead to proper recognition of motion. The other two condition they employed were the omitted wrists and ankles, which allow only the main part of the body to be visible and the figure where shoulders and hips were absent and leave the innermost part of the body invisible.

**FIGURE 1:**
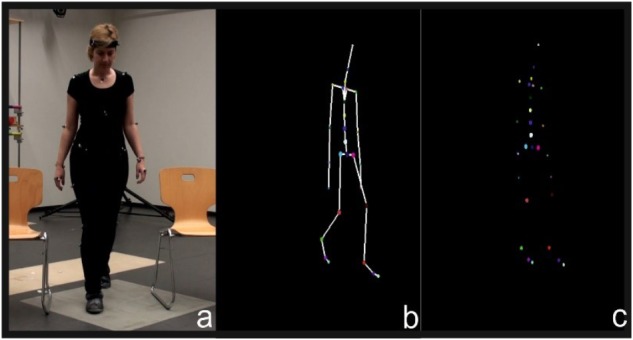
Video, **(a)** avatar with **(b)** bones, point light **(c)** avatar.

According to the second masking technique, either the relevant time or the positions of point lights are disturbed (spatiotemporal jitter), or the body is inverted with the head downward (inversion) (Troje, 2003). These spatiotemporal changes caused the biggest confusion in movement recognition, indicating that neural synapses are activated only when proper time sequence is restored. Hence, vertical spatial placement and temporal succession are fundamental motion perception skills (Blake and Shiffrar, 2007). In addition to spatiotemporal information, research has shown that the perception of motion requires optical attention. Thornton et al. (1999) consider attention to be the cornerstone of human shape recognition, even when continuity of moving point-lights was weak or noisy. Except from adults’ ability to recognize moving light-points based on spatiotemporal information and attention, studies in infants reveal that babies stare point-light sequences, which represent a movement opposed to the point-lights that move randomly (Bertenthal, 1993; Blake and Shiffrar, 2007). In an effort to extend this observation, Pavlova et al., 2002 studied children aged 3–5 years and concluded that they constantly improve their ability to distinguish human shapes appearing with dots. Combining the latest results of Pavlova and the perceived importance of space-time continuum and attention, we conclude that children while improving their skills in dot identification, they also improved the aforementioned skills. In our game, we use avatars constructed by motion captured data and present them as dots, in a similar way as in classic point light display techniques (Figures 1b,c).

### Sensorimotor Collection (Preliminary Study)

Initially, we collected sensorimotor data from native speakers of three languages, in the context of a broader collection related to grounding language to motion, whose subcase is the current tutor. The sensorimotor data collection for the speakers of American English and Greek was conducted at the laboratory of computational vision of Maryland University and for German at the biomechanics laboratory of Excellence Cluster-Cognitive Interaction Technology-University of Bielefeld. In Maryland, we captured our motion data with an Xsens MVN suit, which contains 16 embedded inertial motion detectors. Each sensor consists of 3D gyroscopes, accelerometers and magnetometers. In Bielefeld, we used a Vicon system consisting of 12 cameras, 200 Hz (Figure 1a).

In both cases, we analyzed the sensorimotor data in such a way to recognize latent variables in low level data, which disclose stable patterns in many dimensions. These latent variables appear as distinct groups of synergies and their orientations -as to the axes associated with each joint separately.

The age range of participants is 25–30 years and the gender distribution 5 men and 3 women for each language group. They were chosen very carefully so that all participants were native speakers of each language and were encouraged to perform every prototypical verbal meaning. The sensorimotor collection was divided into two phases: (i) the correspondence between languages and (ii) motion recording.

As soon as we experimentally found the counterpart in different languages, we uttered verbs, such as *walk, trigger, lift, turn, etc*, to participants, who performed the described action. To normalize the process, participants were encouraged to begin moving from a particular angle in a square, for those cases considered it was possible. Basic limitation of the experiment was participants to use only their body and as little as possible items that were considered as strictly necessary for the performance of the verbal concept. Although the majority of actions could be implemented only with the body, there were available objects in the laboratory, such as a plastic step, an inclined plane, one or more tennis balls, a table, a book, a cylinder and a chair. Once again, for normalization purposes, the same objects were available for all sessions, but their use was on the discretion of participants.

The distinctiveness of our dataset lays on the initial design to combine experimentally obtained kinematic data with linguistic knowledge, in order to specify the minimum conceptual representation of a motion event that distinguishes it from all other events. Therefore, the motor data are collected by measuring the performance of native speakers of the above mentioned languages when they are asked to performed the action described by the motion verbs. In the other MoCap databases the performers were acting a series of actions and then the scientists were trimming the data into smaller actions. Our main interest was to see the conceptual representation of each verb, so the participants were asked to perform only what is meant by the verb. Moreover, verbs across languages typologically different are not always accurately translated in WordNet. In our case, we realized difference in action execution between *draskelizo* and *stride*, which appear to be WordNet synonyms. Greek participants made long steps and asked to surpass an obstacle. On the contrary, all English participants made only long steps, while they only associated the obstacle underneath their feet with *step up*. Based on these 3D kinematic data, we reconstructed the avatars that the tutor’s interface exhibits and we will explicitly present them in Section “Architecture of the tutor playing with the stars.”

## A Tutoring System as a Tool for Recognition of Literal Concepts (Study 1)

### Model Architecture of Intelligent Teaching Systems

Considering the pedagogical direction we have set in our research, we combine kinematic data with a diagnostic model, a game-like tutoring system, to help students recognize and differentiate literal from metaphorical meanings. The currently presented pilot study took into account needs and learning problems of students at the primary school level. In the next steps, we will add machine learning modules, which will allow the system to better adapt to each student’s needs.

In general, ITS count more than three decades of design and implementation, while they refer to a plethora of knowledge domains, ranging from science to arts. Carbonell (1970) pioneerly introduced artificial intelligence vision for education as Computer-Assisted Learning, while Sleeman and Brown (1982) named it after the term ITS. According to Murray (2003), an ITS can be built either by using shells or authoring tools regarding the developer’s programming or not background. The shell is a software platform, which offers the necessary components for building an ITS. In most of the cases, the shells provide code libraries, in order to assist the decision making in each module and enable reflection. On the other hand, the authority tools address to non-programmers, such as teachers and instructors, and they are distinguished in pedagogy-oriented or performance oriented tools (Nkambou et al., 2010). The pedagogy-oriented authoring tools focus on organizing both knowledge and goals. The performance-oriented tools emphasize the use of practice and hint giving. Our ITS prioritizes skill acquisition through practice and assistance offering, in case of cognitive deficiency.

A conventional architecture model consists of four structural components (Woolf, 2010):

• Domain knowledge (Woolf, 2010) or cognitive domain model (Freedman, 2000) forms the expert’s knowledge, processes and abilities’ deposit, which serves as the benchmark for students assessment.
• Student knowledge/model contains typical student skills and information concerning misconception or hint giving to current student.
• Tutoring knowledge (Woolf, 2010) or the teaching model (Freedman, 2000) or pedagogical module (Koedinger and Corbett, 2006) includes teaching strategies and methods.
• Communication knowledge (Woolf, 2010) or learning environment (Freedman, 2000) manages user interface and the human-computer communication by employing animated agents, graphic illustrations, etc.

In particular, learning environment determines the activities that will teach problems’ solutions. It usually includes an editor, which can receive text or graphics and shows the student’s responses/movements, while the teaching model is significantly more complex. It supports the process of resolving the problem primarily by checking the correctness of student responses and secondarily by controlling the process through allocation of systematic errors. In case of a wrong answer or systematic errors this pedagogic intelligent tutoring system detects the need for assistance, helps students identify their weakness and indicates hints for self-correcting.

### Assistance for Self-Correcting

An intelligent tutoring system has advanced features to increase productive learning behaviors, supporting both cognitive and metacognitive process. Specifically, cognitive process is subject to the correct ratio between information and assistance offer. McLaren et al. (2008) named this problem as assistance dilemma and stress the need to set detailed parameters. Metacognitive process serves the fundamental principle of learning, namely self-correcting. The main role of providing assistance is to present different strategy to improve self-correcting, such as procedural repetition and socratic dialog (Aleven and Koedinger, 2000).

Concerning the above mentioned parameters of existing ITS, the aroused question is suitable provision of helping. Is it preferable to provide assistance at a student’s request or the system should decide the right time to provide advice? According to Vygotsky’s (1978) theory of higher cognitive functions (1978), since specific learning processes in children profit from the interaction with adults, educators should locate the critical point where a student is no longer able to solve a problem and needs guidance from experts (*zone of proximal development*). In the same line, despite methodological differences between dynamic and interactive assessment (Grigorenko, 2009), share the demand to integrate feedback and learning motives in problem solving tasks. This approach is widely used in several areas such as intelligence tests, action and motor diagnosis (Schack, 2012).

Currently, ITSs give advice mainly after students’ request. According to Baker et al. (2004), there is a possibility that students ‘game’ the system, in order to avoid thinking. So, they prefer quick guess or frequent help request, without prior exhaustion of their cognitive resources. On the other hand, a student may avoid seeking for help when he really needs it. In this point, we come to the second part of our question. How and when can a tutor (computer or human) identify the need for hint giving? The intervention is a difficult task, defined by dialog turns between students and teachers. In the case of our software, we use simpler automatic hint giving techniques such as segmentation of each individual problem and helping at the end of each sub-problem depending on the time of each phase’s completion or failed attempts. Moreover, we incorporate the idea of action support within a zone of proximal development by providing action related prompts and feedback.

An additional parameter for robust hint giving is the different age of students, in relation to that of aforementioned research. Regarding age group, the majority of literature in ITS focused on high school or college students. Piaget (1952) claims that each person can absorb knowledge in a certain way depending on the cognitive age. Primary school children cannot understand every abstract information, since cognitively, they still belong to the stage of concrete operations (7–12 years old). At this stage there is evidence of organized and logical thought, but it has not yet received the abstract form of formal logic, which children will eventually incorporate in the next developmental period (12 years onward). The thought of primary school students is still linked to the tangible reality, so they face difficulties in understanding and transferring knowledge over their mental age. Presumably, based on this point we could explain the discrepancy between Clark and Mayer (2011) and McLaren et al. (2008) concerning personalized principle. According to the first two researchers, informal style can lead to more effective learning compared to official speech in a classroom or in an e-learning environment, while the second experiment showed that the formal language style may be more supportive. In other words, according to Clark and Mayer, instructions and advice should be written in the informal first or second person, while Mclaren, unlike his intuition, claimed that only formal style aligns with cognitive theories of learning. Both approaches can be true depending on the age of the students, given the fact that McLaren’s study involved students who have already obtained Piaget’s highest developmental level. Linguistic style of intervention is of paramount importance regardless of real or virtual classroom environment and is responsible for the success or failure of learning process, depending on several factors, e.g., age. Based on these observations, we use simple and informal style in phrasing the hint, since our target group is elementary school children.

### Tutoring System Enriched With Game-Like Approach

In order to enrich the design of our tool, we incorporate the methodology of (serious) educational games in the student model, which includes a game-like approach of motion and corresponding verbs. Artificial intelligence has long studied individual educational games, which are not embedded in a general educational model. According to review paper Connolly et al. (2012: p. 662, 667), there is lack in coherence and organization in literature, which complicates our understanding of the effects of games in learning. However, concerning learning and behavioral outcome, they found that the most frequent results of games were strong motivation and knowledge acquisition followed by cognitive skills and behavior change, both for entertainment and serious games. Opposite to ordinary games, serious games do not exclusively aim to entertain but to achieve the learning objective, while maintaining a pleasant atmosphere (Houser and DeLoach, 1998). Due to amusement, students interact with the exercises excluding the apprehension of an error, which will likely result in penalties, such as traditional tests (Bizzocchi and Paras, 2005). Moreover, serious educational games fulfill most of the seven conditions of an educational environment.

(1) Motivation.
(2) reinforcement of challenge and undiminished interest, through playful presentation and endogenous fantasy that excites the student.
(3) specific and obvious targets.
(4) high level of interaction and feedback, through hint-giving.
(5) direct occupation with the subject.
(6) evasion of disruptive factors that will divert attention from the subjective experience.
(7) offer of suitable tools to support the above mentioned goals, while the latter two conditions are related solely to software implementation.

### Architecture of the Tutor Playing With the Stars

**FIGURE 2:**
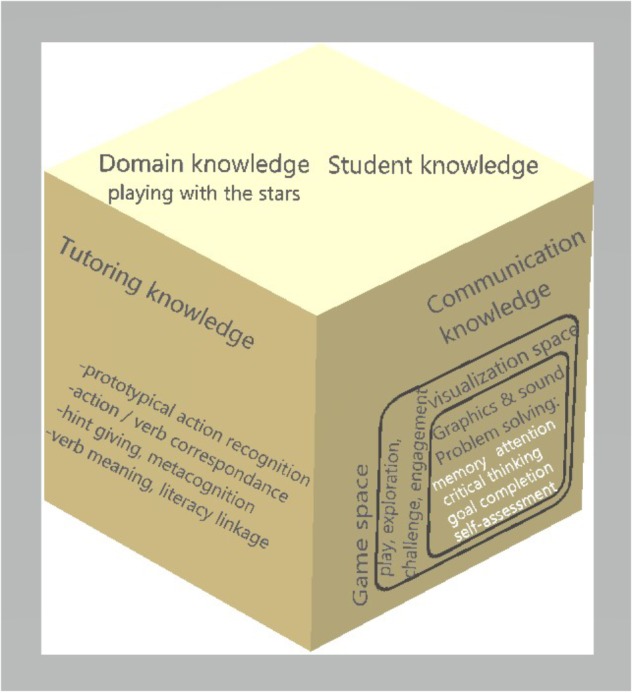
Architecture for the tutor.

In our software’s case, which is called *playing with the stars*, we suggest the following architecture (Figure 2), which combines principles from intelligent teaching systems –our final goal in a later stage, after the addition of machine learning algorithms for better student individualization- and educational games. Therefore, we partially modify the learning environment of Amory et al. (1999), architecture which reinforces the elements of self-correcting and attention, especially for language and movement, while we incorporate it in the overall tutoring scheme. More specifically, our domain knowledge is the playful environment with the stars, the tutoring knowledge consists of the main tutorial goals such as prototypical action recognition, action and verb correspondence and linkage between literacy and metaphora. The communication knowledge is a modification of Amory et al. (1999: p. 319) game and visualization space, in order to link educational with playful elements, a desirable combination for our software. Abstract concepts appear in white fonts -memory attention, critical thinking, goal completion, self- assessment- and promote educational goals while specific notions in black fonts –play, exploration, challenge, engagement- assist in implementing the goals. Both spaces, game and visualization, serve educational elements and problems in a joyful learning environment.

**FIGURE 3:**
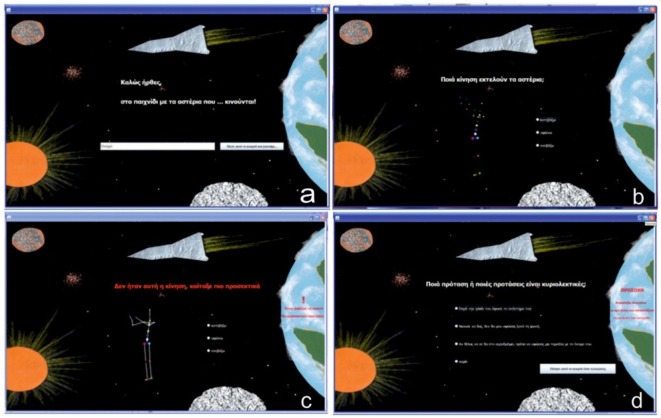
Three levels per verb. **(a)** Welcome page, **(b)** Recognition of the action presented by the “stars”, **(c)** Hint giving page in case of mistake, **(d)** Recognition of literal sentences.

In the opening page, the software is welcoming the student and introduces him to the spectacular scenery of outer space. Planets and point lights, similar to stars, are placed on borderless black background. On this black “sky,” some of the stars will start moving as the avatar’s joint ankles. Avatar’s abstract nature and game’s challenge are the key elements, which cultivate student’s attention and contribute to achieving the educational objectives. The student writes his name and presses the button to proceed level. Verb choices’ and the needed time are recorded in a text file, where the performance scores are gathered (Figure 3a).

On the second page the point light avatar is displayed and the student is asked to recognize the movement and select the correct verb (Figure 3b). In case he fails to select the correct verb, a hint appears. This assistance informs the student for the wrong selection and prompts a better look at the avatar, which in the meanwhile acquires “bones,” i.e., the dots are connected and the human shape is easily distinguished (Figure 3c). Once the action is mapped to the correct verb, the student moves on to identify literal sentences (Figure 3d). He may choose more than one sentences and submit it by pressing the button, regardless of the correctness of his choice. At the same time, a hint is displayed in red color reminding that literal uses are those depicting by the avatar’s moves. So students -who are still in the concrete conceptual stage and gradually construct abstract thinking-can easily and effectively to realize an abstract concept, such as locomotion, if they combine it with concrete representations. When the previous level of correct recognition of literal uses is successfully completed, the process ends with a praise.

## Evaluation of Tutor’s Effectiveness (Study 2)

### Participants

This research tested 69 (*n* = 69) sixth grade students of 6 elementary school classes, all Greek native speakers. Their age ranged from 11–12 years old (*M* = 11.4) and the gender distribution was 38 boys (55%) and 31 girls (45%). We randomly distributed the classes in two experimental groups and one control group. The first experimental group consisted of 24 participants and attended the traditional approach (TG), while the second one had 25 participants interacting with the game-like intelligent tutoring system (GG). In the control group (CG) we assigned 20 students. The inventions were held in the everyday school program for 4 weeks, during a 2-h per week lesson, which allows teachers to involve their classes in educational projects according to their will or interest. Prior to data analysis, we obtained consent forms from parents, in order to anonymously use the performance results.

### Procedure

Diagnostical pre-tests were conducted on the first week for all the groups. The pre-test was a written questionnaire where each question stated a verb and it was followed by three sentences, e.g., for the verb *σηκώνω* (lift):

(1) *Bαρέθηκα να σηκώνω τα βάρη της* ο*ικ*ο *γ ένειας*
I am bored to lift family weights = undertake (metaphor)
(2)
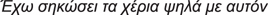
I have lifted my arms high due to him = surrendered (metaphor)
(3)
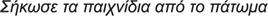
He lifted the toys from the floor (literal)

**FIGURE 4:**
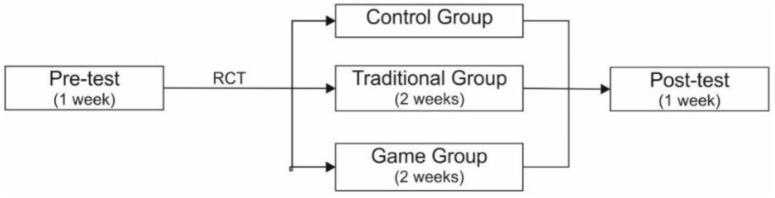
Schematic study design.

Students were asked to recognize whether each sentence was literal or metaphorical. None of the participants had been taught the distinction between linguistic literacy and metaphor in sixth grade, even though they were exposed to the phenomenon in previous grades, according to the spiral curriculum, where the same phenomenon is taught in different grades starting from general approach toward complex and overall presentation (Barnes, 1991). The control group did not receive any intervention for the next 2 weeks and students were only asked to read literature. The same period, the experimental groups (TG, GG) were receiving the interventions, while by the end of the 4th week, all groups did the post-test assessments (Figure 4). Written or digital data were anonymous and analyzed by the tutors that did the interventions, based on whether the students had correctly recognized literal from metaphorical sentences.

**FIGURE 5:**
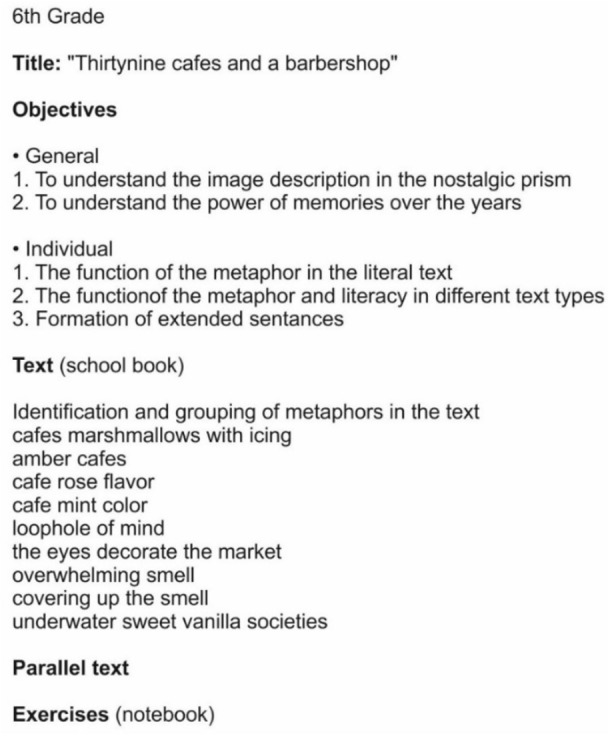
Traditional approach.

Concerning interventions, the experimental group TG complied with the curriculum (Figure 5). TG was taught the course “thirty nine coffee shops and a barber” from the student’s book following a traditional work plan (Figure 4), which consists of traditional teaching with the help of text, blackboard and written homework. The experimental group GG was asked to interact with the ITS “playing with stars” as described at Section “Assistance for self-correcting.”

### Data Analysis

The experimental study employs pre and post-tests in a control group design. For the distribution of the school classes, we followed randomized control trial (RCT), while the participants had been randomly assigned to each class during the creation procedure of the class for the first grade. In this way, classes exhibit similar variation in learning achievements. Concerning statistical analysis, we test the first hypothesis with a one way analysis of variance (ANOVA), in order to understand if there is any significant difference between the three groups’ pre-tests. For the second hypothesis, we conducted a two way analysis of variance with repeated measures (alpha = 0.05) to evaluate the improvement in scores of GG and TG compared to those of the control group. Within subjects variable is *test* with two levels (pre and post-test performance) and between subjects is the variable *group* with three levels (GG, TG, and CG). The main effect was tested with *Wilks’ Lambda* and the effects size was calculated with *eta squared* (η^2^) (similar to Vernadakis et al., 2015). Furthermore, we estimated a one way ANOVA with *Scheffe*’s *post hoc* analysis with dependent variable the *improvement* between pre and post-test and independent variable *groups* with three levels (GG, TG, and CG). Finally, in order to evaluate the third hypothesis, we analyze a *t*-test with independent variables the *metacognitive* performance of TG and students.

The hypotheses are concretely presented as follows:

H_1_. The three groups (CG, TG, GG) will not differ at pre-test.
H_2_. The students in the two experimental groups improve compared to those in CG, while we expect that GG participants will show higher learning achievements than TG.
H_3_. The students in GG will exhibit gradual improvement in the duration of action recognition in the experimental procedure, which implies improvement in attention and spatiotemporal memory.

## Results

The first hypothesis assumed that the mean score of mistakes per question in the three groups for the pre-test would not differ significantly. H_1_ was evaluated with a one way analysis of variance (ANOVA) and corroborated. There was no significant difference between the three groups at the beginning of the experiment, *F*(2,66) = 1.09, *p* = 0.341.

**FIGURE 6:**
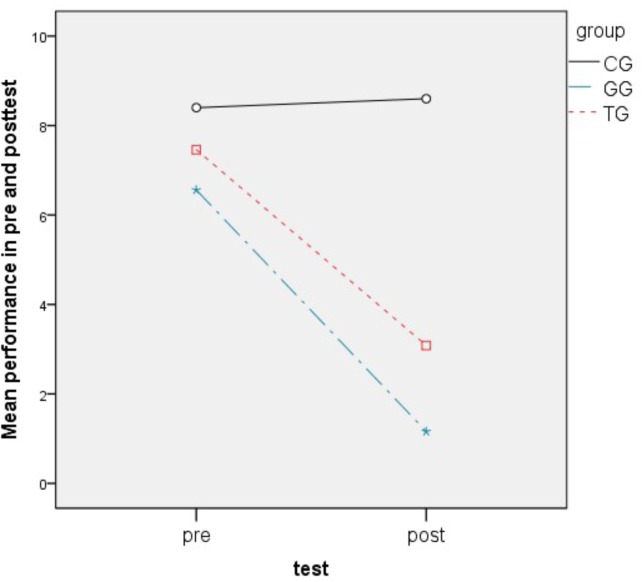
Performance of the three group in pre and post-tests.

**FIGURE 7:**
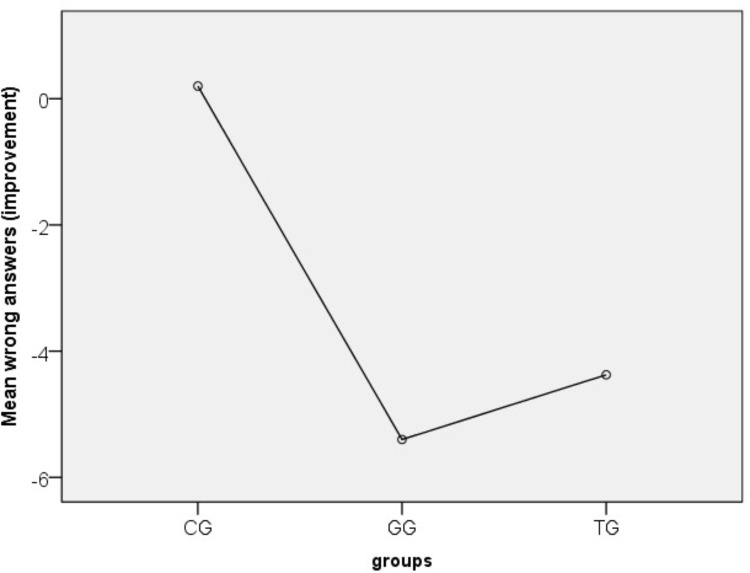
Performance in post-test for the three groups.

The second research hypothesis (H_2_) compares learning achievements across groups. At first we analyzed a two way ANOVA with repeated measurements in order to define if different groups’ approaches influence the performance across time. Variable *tests* had significant effect according to *Wilks’ Lambda* = 0.364, *F*(1,66) = 115.23, *p* = 0.000 with partial η^2^ = 0.636, which can be interpreted as significant difference between pre an post-tests in general, while the interaction between *tests x groups* allowed us to show that the groups have significant changes across tests relatively to each other with *Wilks’ Lambda* = 0.489, *F*(2,66) = 31.6, *p* = 0.000 with partial η^2^ = 0.489 Coherent is the between subjects result of *groups* (*p* = 0.000) when groups significantly differ even when pre and post-tests are combined. In order to particularize the improvement in the groups, we calculated the possible *improvement* between tests and used it as dependent variable in one way ANOVA with *Scheffe post hoc* adjustments, while the independent variable was *groups*. *Scheffe post hoc* test shows that the mean improvement of CG was significantly different then TG (*M* = 4.57, *p* = 0.000) or GG (*M* = 5.60, *p* = 0.000). If we take into account Figure 6, we can clearly see that CG has shown no improvement compared to GG and TG. Moreover, GG participants succeeded less wrong answers in the linguistic task and higher learning achievements (*M* = 4.4, *SD* = 2.4, 95% CI:3.4 to 5.4, *p* = 0.000) compared to TG participants (*M* = 5.4, *SD* = 2.9, 95% CI:4.2 to 6.6, *p* = 0.000) (Figure 7).

**FIGURE 8:**
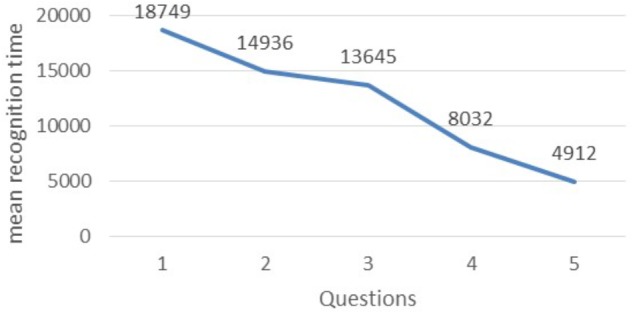
Mean recognition time.

**FIGURE 9:**
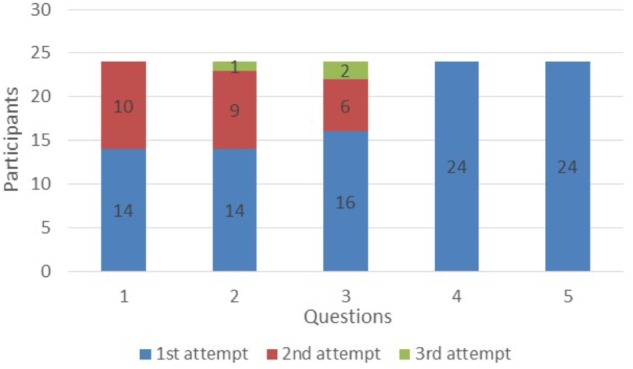
Attemps to recognize the action.

Concerning the third hypothesis (H_3_), we notice an improvement in mean duration action recognition in the GG (Figures 8, 9). At the first question students needed approximately 19 s to identify the action performed by the stars. The time was gradually decreased and finally at the last 2 questions they needed between 8 and 5 s.

## Discussion

Our software’s pedagogical objectives are to familiarize students with prototypical actions to better understand-declare literal language, sharpen students’ spatiotemporal memory and increase their attention. The latter two aims are inherent in the abstract presentation of the kinetic data (avatar) and in consistent identification of motor patterns.

The thematic goal can vary between interdisciplinary teaching projects as purely linguistic problems. Considering the age of the students and the instructions of curricula, we chose to investigate the procedure of consistent distinction between literal from non-literal motion verbs’ uses. According to literature, (Asch and Nerlove, 1960; Vosniadou, 1987; Schecter and Broughton, 1991; Selimis, 2007 for the review) the ability to understand metaphor grows faster compared to other cognitive functions even in very young age. Thus, in our groups’ written diagnostic pre-tests (H_1_), we identify that students at the age of 11 years face difficulties in recognizing literal from metaphorical sentences. Even though, researchers do not fully agree on the terminal age that metaphor is fully understood, they comply that the conquest of literal meanings precedes and constitutes the basic ability to build the opposite concept of non-literal/metaphorical notions. Using this reasoning as starting point for our intelligent system, we present the prototypical-representative depiction of every movement and offer feedback to students in order to recognize the literal use of the verb, which corresponds to the avatar movement, which they have just seen. After that, students have been able to easier identify the metacognitive mechanism, namely the connection between literal verb use and prototypical action, than in traditional class, where the same mechanism was only verbally explained but not concretely and experientially presented. Moreover, the phraseology that students used to describe why a verb’s use is non-literal, strongly reminds the short hint that appeared during the game, in case of mistake.

Furthermore, the significant better results of the game group and the particular sensory kinetic functions of the software naturally guide students, who are absorbed by playful stream and visualizations of each verb. Thereby, it ensures a simple process unlike the demanding procedures used in most of the language teaching diagnostic tests.

Concerning improvement of spatiotemporal memory and increased attention, we used the prevailing conceptualization of the whole body movement in boundless space. This design utilizes Papafragou and Selimis’s (2007) findings for the conceptualization of abstract phenomena through movement. These phenomena can be either articles or locations. In our case, point-light stars placed on the abstract background of outer space serve children’s natural selection to identify the uncontrolled state as an entity that generally moves in any space or a move toward a location, which subliminary has limits. The abstract nature of this acting avatar and the inherent challenge of a game set-up, optimizes attention and contribute to achieve spatial perception. Finally, gradual elimination of the time needed for cognition of the action indicates possible improvement in the (spatial) temporal memory, as well, but we intend to test it with functional methods, such as EEG and structural dimensional analysis (Schack, 2012) in a similar set up.

## Conclusion

Even though, the current tutoring system is a pilot study, which incorporates sensorimotor data and linguistic knowledge, it elicits language related learning achievements and at the same time –based on first indications of reduced time for avatar recognition- might boost spatiotemporal memory, attention and through hint-providing cultivates better metacognitive mechanisms, which can help elementary school students to overcome cognitive limitations of their age urging them to abstract reasoning.

Traditional teaching approaches, such as the use of textbooks, homework, and blackboard, have been extensively tested in the past and enriched with diverse intervention tools. In abstract, almost literature metaphors, only texts can provide necessary stylistic information that will elevate cognitive concepts and their linguistic expressions. Thus, our data and analysis show that a game-like approach like virtual reality environment could benefit traditional interventions, especially for elementary school students. Furthermore, our study introduces the use of kinetic data by deploying embodiment for the needs of language learning, since based on the primary sensorimotor patterns helps in association with abstract concepts. In the next step, we will further enrich the tutor with machine learning techniques to permit adaptiveness to student’s needs and run more detailed memory related measurements, which enable us to deeper understand the mechanism of spatiotemporal memory. Finally, we will expand the topic to other language education problem, e.g., foreign language learning.

## Ethics Statement

This study was carried out in accordance with the recommendations of the Greek Ministry of Education, which approved the protocol. All subjects’ parents gave written informed consent in accordance with the Declaration of Helsinki.

## Author Contributions

All authors made substantial contribution to the design of this work, acquisition, analysis, or interpretation of data for the work and agreed to be accountable for all aspects of the work in ensuring that questions related to the accuracy or integrity of any part of the work are appropriately investigated and resolved. MS and YA collaborated on the American English and Greek sensorimotor data design, collection, and analysis. MS and TS worked together for the German 3D data collection and analysis. MS, TS, and YA contributed to the final manuscript.

## Conflict of Interest Statement

The authors declare that the research was conducted in the absence of any commercial or financial relationships that could be construed as a potential conflict of interest.
